# Evaluation of Data Sharing After Implementation of the International Committee of Medical Journal Editors Data Sharing Statement Requirement

**DOI:** 10.1001/jamanetworkopen.2020.33972

**Published:** 2021-01-28

**Authors:** Valentin Danchev, Yan Min, John Borghi, Mike Baiocchi, John P. A. Ioannidis

**Affiliations:** 1 Meta-Research Innovation Center at Stanford, Stanford University School of Medicine, Stanford, California; 2 Stanford Prevention Research Center, Department of Medicine, Stanford University School of Medicine, Stanford, California; 3 Now with Department of Sociology, University of Essex, Colchester, United Kingdom; 4 Department of Epidemiology and Population Health, Stanford University School of Medicine, Stanford, California; 5 Lane Medical Library, Stanford University School of Medicine, Stanford, California; 6 Department of Biomedical Data Science, Stanford University School of Medicine, Stanford, California

## Abstract

**Importance:**

The benefits of responsible sharing of individual-participant data (IPD) from clinical studies are well recognized, but stakeholders often disagree on how to align those benefits with privacy risks, costs, and incentives for clinical trialists and sponsors. The International Committee of Medical Journal Editors (ICMJE) required a data sharing statement (DSS) from submissions reporting clinical trials effective July 1, 2018. The required DSSs provide a window into current data sharing rates, practices, and norms among trialists and sponsors.

**Objective:**

To evaluate the implementation of the ICMJE DSS requirement in 3 leading medical journals: *JAMA*, *Lancet*, and *New England Journal of Medicine* (*NEJM*).

**Design, Setting, and Participants:**

This is a cross-sectional study of clinical trial reports published as articles in *JAMA*, *Lancet*, and *NEJM* between July 1, 2018, and April 4, 2020. Articles not eligible for DSS, including observational studies and letters or correspondence, were excluded. A MEDLINE/PubMed search identified 487 eligible clinical trials in *JAMA* (112 trials), *Lancet* (147 trials), and *NEJM* (228 trials). Two reviewers evaluated each of the 487 articles independently.

**Exposure:**

Publication of clinical trial reports in an ICMJE medical journal requiring a DSS.

**Main Outcomes and Measures:**

The primary outcomes of the study were declared data availability and actual data availability in repositories. Other captured outcomes were data type, access, and conditions and reasons for data availability or unavailability. Associations with funding sources were examined.

**Results:**

A total of 334 of 487 articles (68.6%; 95% CI, 64%-73%) declared data sharing, with nonindustry NIH-funded trials exhibiting the highest rates of declared data sharing (89%; 95% CI, 80%-98%) and industry-funded trials the lowest (61%; 95% CI, 54%-68%). However, only 2 IPD sets (0.6%; 95% CI, 0.0%-1.5%) were actually deidentified and publicly available as of April 10, 2020. The remaining were supposedly accessible via request to authors (143 of 334 articles [42.8%]), repository (89 of 334 articles [26.6%]), and company (78 of 334 articles [23.4%]). Among the 89 articles declaring that IPD would be stored in repositories, only 17 (19.1%) deposited data, mostly because of embargo and regulatory approval. Embargo was set in 47.3% of data-sharing articles (158 of 334), and in half of them the period exceeded 1 year or was unspecified.

**Conclusions and Relevance:**

Most trials published in *JAMA*, *Lancet*, and *NEJM* after the implementation of the ICMJE policy declared their intent to make clinical data available. However, a wide gap between declared and actual data sharing exists. To improve transparency and data reuse, journals should promote the use of unique pointers to data set location and standardized choices for embargo periods and access requirements.

## Key Points

**Question:**

What are the rates of declared and actual sharing of clinical trial data after the medical journals’ implementation of the International Committee of Medical Journal Editors data sharing statement requirement?

**Findings:**

In this cross-sectional study of 487 clinical trials published in *JAMA*, *Lancet*, and *New England Journal of Medicine*, 334 articles (68.6%) declared data sharing. Only 2 (0.6%) individual-participant data sets were actually deidentified and publicly available on a journal website, and among the 89 articles declaring that individual-participant data would be stored in secure repositories, data from only 17 articles were found in the respective repositories as of April 10, 2020.

**Meaning:**

These findings suggest that there is a wide gap between declared and actual sharing of clinical trial data.

## Teaser

This cross-sectional study evaluates the implementation of the International Committee of Medical Journal Editors (ICMJE) data sharing statement requirement in 3 leading medical journals.

## Introduction

Responsible sharing of individual-participant data (IPD) from clinical studies has gained increasing traction and has been advocated for many years by many scientists and scientific leadership organizations.^1^ However, promoting data sharing from clinical trials has not been straightforward, and there has been much debate surrounding privacy risks and the optimal incentives for clinical trialists and sponsors.^2,3,4,5,6^ Recently, the International Committee of Medical Journal Editors (ICMJE) implemented a clinical trial data sharing policy. The policy does not mandate^7,8^ data sharing but requires a data sharing statement (DSS) from submissions reporting clinical trials effective July 1, 2018.^9,10,11^

Prior work has identified a range of potential risks preventing trialists from sharing IPD.^3,12,13^ These risks include protection of patient privacy and confidentiality,^4,13^ inappropriate data reuse and replication,^12^ and researchers’ and sponsors’ potential losses of secondary publications and product advantage, respectively, because of the use of the shared data by competitors.^3,5,14^ Repositories for clinical data from industry-funded^15,16,17^ and publicly funded^18^ trials have provided a safeguarded mechanism for responsible IPD sharing, thereby substantially minimizing patient privacy and confidentiality risks. However, perceived risks of inappropriate reuse and competition have been difficult to mitigate, especially when the current reward system for researchers predominantly incentivizes high-impact publications, often based on exclusive data, at the expense of transparency, reproducibility, and data reuse.^19,20,21,22^

Disincentives for data sharing are known to have a disproportionate impact on clinical studies because the process of conducting those studies is time, cost, and labor intensive.^3^ Yet the role of prevalent disincentives and incentives (eg, data authorship^23,24^) for clinical trial data sharing have only recently entered the public realm,^3,23,25,26^ in part accelerated by discussions surrounding the ICMJE’s data sharing policy^11,27^ when many points of agreement and disagreement among stakeholders were articulated.^3,5,6,27,28^

Many data repositories have been established to facilitate secure sharing of IPD from clinical trials.^15,17,18,29,30,31,32^ Some industry sponsors, such as GlaxoSmithKline and Johnson & Johnson, have initiated their own data sharing repositories and partnerships with ClinicalStudyDataRequest.com (CSDR)^17^ and the Yale University Open Data Access (YODA) Project,^15^ respectively. Foundations and research charities, such as Wellcome Trust, Cancer Research UK, the Bill and Melinda Gates Foundation, and the UK Medical Research Council, have also implemented clinical trial data sharing policies and are now members of the CSDR platform.^5,33^ Information on CSDR, the YODA Project, and other clinical trial data repositories can be found in the eTable in the Supplement.

Following the 2003 Data Sharing Policy^34^ that encouraged data plan and sharing of grants exceeding $500 000, the National Institutes of Health (NIH) have implemented disease-specific data repositories, including the Biologic Specimen and Data Repository Information Coordinating Center,^18^ which is supported by the National Heart, Lung, and Blood Institute (NHLBI), and the NCTN/NCORP Data Archive for data sets from clinical trials of the National Clinical Trials Network (NCTN) and the NCI Community Oncology Research Program (NCORP), which is supported by the National Cancer Institute. Recently, the NIH announced new Policy for Data Management and Sharing^35^ with direct implications for clinical trial data sharing.^21^

Prior research has identified differences in clinical trial data sharing across funders and sponsors.^30,36,37^ Historically, sharing of clinical trial data has been more prevailing among industry funders and sponsors associated with data repositories. For example, for a subset of drugs and industry sponsors (ie, Roche, Lilly, Boehringer Ingelheim, and GlaxoSmithKline), Boutron et al^30^ found that 53% (512 of 966 randomized clinical trials) of clinical trials registered on ClinicalTrials.gov were listed on CSDR, and for 40% (385 of 966 randomized clinical trials) of the trials all documents were available, including raw data sets. Academic research has been less engaged with clinical trial data sharing. For instance, a prominent NIH repository such as the NHLBI Data Repository contained only 100 studies as of May 31, 2016.^18^ However, a study^37^ of trialists’ intentions to share IPD from clinical trials registered on ClinicalTrials.gov between January 2016 and August 2017 found that NIH-funded trials were more likely to indicate data sharing intentions than industry-funded trials.

The ICMJE policy requires investigators to state whether they will share data (or not) while simultaneously providing an opportunity for them to place multiple restrictions and conditions regarding data access. Specifically, the DSS provides an opportunity for authors and sponsors to specify periods of data exclusivity or embargo. In addition, authors can specify in the DSS how the data will be made available, reasons for data availability or unavailability, and related preferences (for examples of DSSs, see eAppendix 1 in the Supplement). Thus, the DSSs, required by the ICMJE’s policy, provide a window into data sharing norms, practices, and perceived risks among trialists and sponsors. We set out to evaluate how the ICMJE’s data sharing policy has been implemented in 3 leading medical journals that are also member journals of ICMJE: *JAMA*,^9^ *Lancet*,^10^ and *New England Journal of Medicine* (*NEJM*).^11^

## Methods

Because this study used publicly available data and did not involve human participants, institutional review board approval and informed consent were not sought, in accordance with 45 CFR §46. The Strengthening the Reporting of Observational Studies in Epidemiology (STROBE) reporting guideline^38^ was used as a guideline for this observational study. In particular, the STROBE checklist for cross-sectional studies was used to ensure accurate reporting.

The inclusion criteria in this study were reports of clinical trials, published as articles in *JAMA*, *Lancet*, and *NEJM* between July 1, 2018, and April 4, 2020, and containing a DSS. Excluded from the study were publications that contained no DSS because the article was submitted prior to July 1, 2018; was an observational or other type of study that is different from clinical trial and is, therefore, not required to contain a DSS under the ICMJE policy; or was a letter, correspondence, or other type of publication that lacks a DSS.

A MEDLINE/PubMed search of clinical trials published in the 3 journals between July 1, 2018, and April 4, 2020, identified 629 potentially eligible articles; 486 of them included a DSS, whereas the others were either submitted before July 2018, were not a clinical trial, or were letters. One article published in 2020 met all other inclusion criteria but contained no DSS and was included in the study sample as not sharing data because articles published in 2020 were likely submitted after July 1, 2018, and were, therefore, required to contain a DSS. We conducted a cross-sectional observational study for all 487 articles in *JAMA* (112 articles), *Lancet* (147 articles), and *NEJM* (228 articles) (eFigure in the Supplement). Two reviewers evaluated each article independently. Discrepancies were resolved unanimously or by a third reviewer.

Data were classified as being available when authors answered yes (*JAMA* and *NEJM*) or gave an unstructured positive response (*Lancet*) to the data availability question. Information about data type, access, conditions, and reasons for data availability or unavailability were taken from the DSS (eAppendix 2 in the Supplement). We also compared declared with actual data availability in repositories by examining whether information about data and data themselves are available in the respective repository. For more details on the MEDLINE/PubMed search strategy, inclusion and exclusion criteria for the study, definition of variables, and data extraction, see eAppendix 3 in the Supplement.

Drawing on prior research reporting differences in intention to share clinical trial data between industry-funded and nonindustry (including NIH)–funded clinical trials,^37^ we classified funding sources as industry, nonindustry NIH, nonindustry non-NIH, and mixed. Industry refers to research funding from companies. Nonindustry NIH refers to research funding from the US NIH. Nonindustry non-NIH refers to research funding from foundations, trusts, associations, national institutes outside the US, and so forth. Mixed refers to any combination of the other research-funding categories.

### Statistical Analysis

We conducted a descriptive analysis of variables associated with data sharing by type of funding and publication journal. For the primary outcome variables, declared and actual data sharing, we report the 95% CIs determined by bootstrapping (100 000 iterations). The χ^2^ test was used for comparing differences in prevalence of declared data sharing between types of funding. The 2-tailed Fisher exact test was used for comparing differences in prevalence of data availability in repositories between types of funding. *P* < .05 was considered significant. To perform data analysis and to generate summary statistics and graphs, we used the Python programming language version 3.8.3 (Python Software Foundation), a Jupyter Notebook,^39^ and the following libraries: SciPy,^40^ Pandas,^41^ NumPy,^42^ Matplotlib,^43^ Scikits-Bootstrap,^44^ and Seaborn.^45^ For the statistical tests, we used the Python package Statsmodels and R statistical software version 4.0.2 (R Project for Statistical Computing).

## Results

**Figure 1.**
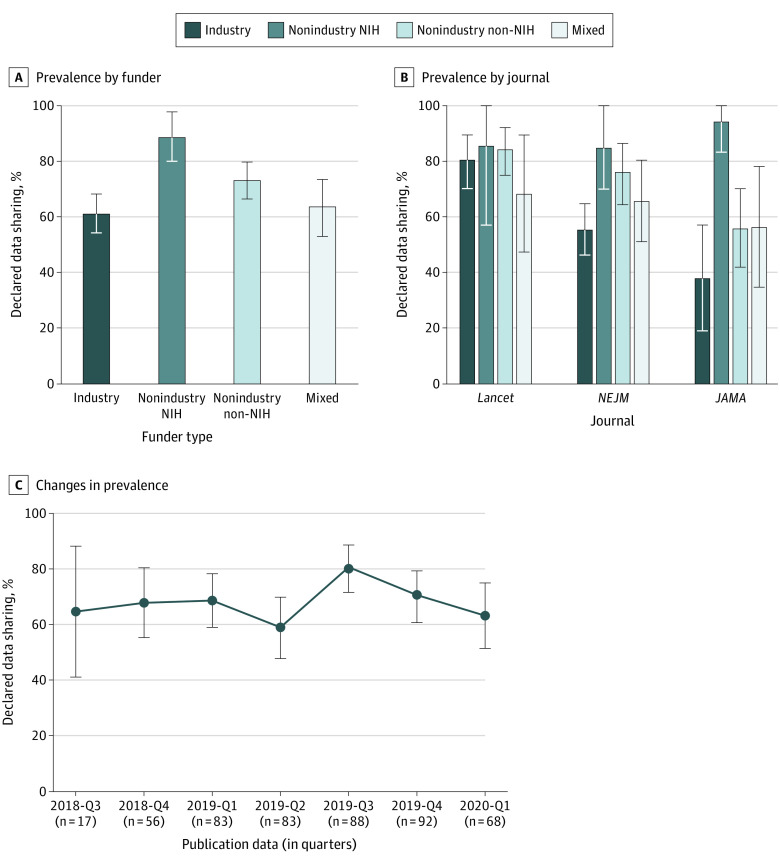
Declared Clinical Trial Data Sharing in 3 Leading Medical Journals Graphs show prevalence of data sharing by funder (A) and journal (B) and changes in prevalence between 2018 and 2020 (C). Error bars denote 95% CIs. *NEJM* indicates *New England Journal of Medicine*; NIH, National Institutes of Health.

Overall, 334 of 487 articles (68.6%; 95% CI, 64%-73%) declared data sharing (Table 1). The prevalence of declared data sharing varied by funder type for all 487 articles (χ^2^_3_ = 15.93; *P* = .001). Nonindustry NIH-funded trials had the highest rates of declared data sharing (89%; 95% CI, 80%-98%) and industry-funded trials had the lowest rates (61%; 95% CI, 54%-68%) (Figure 1A). The ranking of funders regarding declared data sharing is largely consistent across the 3 journals—NIH, nonindustry non-NIH, mixed, and industry—although the 95% CIs overlap (Figure 1B). No substantial changes in the prevalence of declared data sharing were observed over the span of the first 7 quarters of policy implementation (Figure 1C).

**Table 1.** Prevalence and Conditions of Declared Clinical Trial Data Sharing by Type of Funding

Variable	Articles, No./Total (%)				
	Industry (n = 186)	Nonindustry (n = 45)	Nonindustry, non-NIH (n = 173)	Mixed (n = 83)	Total (N = 487)
Declared data sharing, articles, No. (%)^a^					
Yes	114 (61.3)	40 (88.9)	127 (73.4)	53 (63.9)	334 (68.6)
No	72 (38.7)	5 (11.1)	46 (26.6)	30 (36.1)	153 (31.4)
Journal, articles, No. (%)					
* JAMA*	21 (11.3)	18 (40.0)	50 (28.9)	23 (27.7)	112 (23.0)
* Lancet*	57 (30.6)	7 (15.6)	64 (37.0)	19 (22.9)	147 (30.2)
* New England Journal of Medicine*	108 (58.1)	20 (44.4)	59 (34.1)	41 (49.4)	228 (46.8)
Type of declared available data^b^					
Deidentified individual-participant data	83/114 (72.8)	33/40 (82.5)	95/127 (74.8)	44/53 (83.0)	255/334 (76.3)
Aggregate data only	2/114 (1.8)	0/40 (0.0)	1/127 (0.8)	0/53 (0.0)	3/334 (0.9)
Unspecified or partial data^c^	29/114 (25.4)	7/40 (17.5)	31/127 (24.4)	9/53 (17.0)	76/334 (22.8)
Access to data^d^					
Request to authors	9/114 (7.9)	15/40 (37.5)	96/127 (75.6)	23/53 (43.4)	143/334 (42.8)
Request to committee, group, or unit	15/114 (13.2)	3/40 (7.5)	36/127 (28.3)	13/53 (24.5)	67/334 (20.1)
Request to repository or archive	46/114 (40.4)	22/40 (55.0)	5/127 (3.9)	16/53 (30.2)	89/334 (26.6)
Request to company	72/114 (63.2)	0/40 (0.0)	0/127 (0.0)	6/53 (11.3)	78/334 (23.4)
Access unspecified	5/114 (4.4)	2/40 (5.0)	5/127 (3.9)	3/53 (5.7)	15/334 (4.5)
Data are available to others	0/114 (0.0)	0/40 (0.0)	2/127 (1.6)	0/53 (0.0)	2/334 (0.6)
Conditional data access^d^					
Data embargo	49/114 (43.0)	22/40 (55.0)	57/127 (44.9)	30/53 (56.6)	158/334 (47.3)
Up to 1 y^e^	20/114 (17.5)	13/40 (32.5)	30/127 (23.6)	18/53 (34.0)	81/334 (24.3)
>1 y to 2 y	13/114 (11.4)	7/40 (17.5)	13/127 (10.2)	6/53 (11.3)	39/334 (11.7)
>2 y	4/114 (3.5)	2/40 (5.0)	4/127 (3.1)	5/53 (9.4)	15/334 (4.5)
Product approval	36/114 (31.6)	0/40 (0.0)	0/127 (0.0)	1/53 (1.9)	37/334 (11.1)
Collaboration	0/114 (0.0)	2/40 (5.0)	6/127(4.7)	1/53 (1.9)	9/334 (2.7)
Reasons for why data not available^d^					
No reason given	40/72 (55.6)	2/5 (40.0)	26/46 (56.5)	15/30 (50.0)	83/153 (54.2)
Data privacy	3/72 (4.2)	1/5 (20.0)	9/46 (19.6)	2/30 (6.7)	15/153 (9.8)
Time and cost	0/72 (0.0)	0/5 (0.0)	2/46 (4.3)	0/30 (0.0)	2/153 (1.3)
Ongoing trial or research	2/72 (2.8)	0/5 (0.0)	2/46 (4.3)	9/30 (30.0)	13/153 (8.5)
Regulatory approval	3/72 (4.2)	0/5 (0.0)	1/46 (2.2)	0/30 (0.0)	4/153 (2.6)
Proprietary data	7/72 (9.7)	0/5 (0.0)	4/46 (8.7)	3/30 (10.0)	14/153 (9.2)
Shared among coinvestigators only	2/72 (2.8)	0/5 (0.0)	0/46 (0.0)	0/30 (0.0)	2/153 (1.3)
Data may be available for collaboration	6/72 (8.3)	0/5 (0.0)	0/46 (0.0)	1/30 (3.3)	7/153 (4.6)
Data may be available upon request	19/72 (26.4)	2/5 (40.0)	6/46 (13.0)	7/30 (23.3)	34/153 (22.2)

^a^ The denominators represent the total for the respective column, unless otherwise indicated.

^b^ Detailed definitions of variables are provided in eAppendix 2 in the Supplement.

^c^ Among the articles not specifying the type of data, 13 intended to store data in clinical trial data repositories, presumably individual-participant data, and 1 made available (via collaboration) individual-participant data in a designated repository.

^d^ Categories are not mutually exclusive.

^e^ Time periods are not specified in all articles proposing data embargo.

The presence of multiple articles from the same clinical trials would violate the assumption of independence and could also introduce social dependencies,^46^ such as clustering by authors, funder, or institution. To address this issue, we identified 12 clusters of multiple publications for the same trial. Each cluster contained 2 or (in 1 instance) 3 articles that had the same declared data sharing and funding source and were associated with common authors and institutions. We treated each cluster as a single article observation (474 articles) and recomputed our results about declared data sharing by funding as a way of assessing the impact of clustering on our results. Results were qualitatively similar: 326 of 474 articles declared data sharing (68.8%; 95% CI, 64%-73%), and differences in declared data sharing by funding sources were similar to the ones in the entire data set: NIH (88.4%; 95% CI, 79%-98%), nonindustry non-NIH (73.5%; 95% CI, 67%-80%), mixed (65.4%; 95% CI, 55%-76%), and industry (61.2%; 95% CI, 54%-68%). Owing to the small effect of clustering of articles from the same trial, the subsequent analysis uses the entire data set of 487 articles.

Industry sponsors had the lowest rate of declared data sharing in our sample. To examine further, we sampled industry sponsors that are members of the largest industry-initiated registry, CSDR, as of November 8, 2020, and identified the following industry sponsors with 1 or more studies in our sample: GlaxoSmithKline (10 studies), Novartis (10 studies), Bayer (6 studies), ViiV Healthcare (3 studies), ONO Pharmaceutical (2 studies), Sanofi (2 studies), Eisai (2 studies), Astellas (1 study), and Chugai (1 study). All of those industry sponsors are listed as current members of CSDR; industry sponsors that declared in their DSS that they would deposit data in CSDR but were not listed as members of the registry were excluded from this analysis. The rate of declared data sharing for all industry members of CSDR (56.8%; 95% CI, 38%-70%) was similar to the rate we established for all industry-funded trials (61.3%; 95% CI, 54%-68%). We could, therefore, exclude the possibility that the lowest rate of declared data sharing of industry funders is due to differences between companies that are members of data repositories and companies that are not members of data repositories.

Regarding type of shared data, 76.3% (255 of 334) of the articles proposed to provide deidentified IPD, 22.8% (76 of 334) would provide unspecified or partial data, and 0.9% (3 of 334) would provide aggregate data only. Only 2 of 334 IPD sets (0.6%; 95% CI, 0.0%-1.5%) were actually deidentified and publicly available (on journal website) as of April 10, 2020 (both data sets were associated with the same clinical trial). The remaining were supposedly accessible via request to authors (143 of 334 articles [42.8%]), repository (89 of 334 articles [26.6%]), and company (78 of 334 articles [23.4%] overall; 72 of 114 articles [63.2%] among industry-funded trials).

Conditions for access to data included embargo (158 of 334 articles [47.3%]), product approval (37 of 334 articles [11.1%]), and collaboration (9 of 334 articles [2.7%]). Among the 158 articles specifying embargo, approximately one-half required 1 year or less of data exclusivity. In the other half of embargo cases, the embargo period exceeded 1 year or was unspecified.

**Figure 2.**
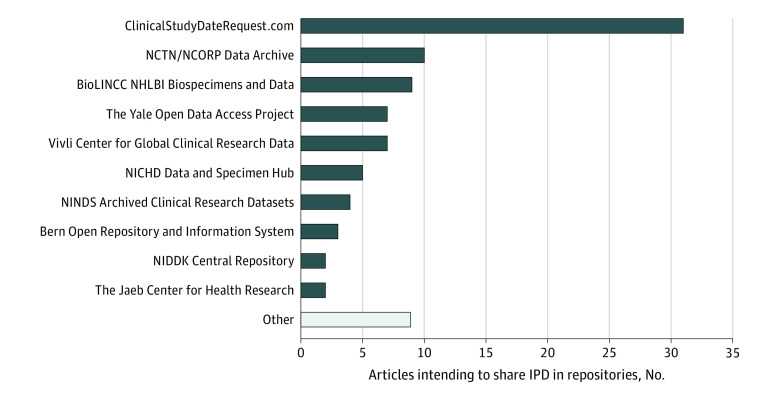
Ranking of Data Repositories by the Number of Articles Intending to Share Individual-Participant Data (IPD) in the Respective Repository BioLINCC indicates Biologic Specimen and Data Repository Information Coordinating Center; NCORP, National Cancer Institute Community Oncology Research Program; NCTN, National Clinical Trials Network; NHLBI, National Heart, Lung, and Blood Institute; NICHD, National Institute of Child Health and Human Development; NIDDK, National Institute of Diabetes and Digestive and Kidney Diseases; and NINDS, National Institute of Neurological Disorders and Stroke.

Data repositories have a central role in improving sharing, security, discoverability, and reuse of research data,^47,48^ particularly IPD from clinical trials.^29,36,49^ Among the 89 articles proposing to make IPD available through repositories, many planned to store data in general-purpose repositories, including the CSDR (31 articles), the YODA Project (7 articles), and Vivli (7 articles). Another 30 articles planned to store IPD in NIH-supported, domain-specific data repositories, such as the NCTN/NCORP Data Archive (10 articles), the NHLBI Biologic Specimen and Data Repository Information Coordinating Center (9 articles), and the National Institute of Child Health and Human Development Data and Specimen Hub (5 articles) (Figure 2).

**Figure 3.**
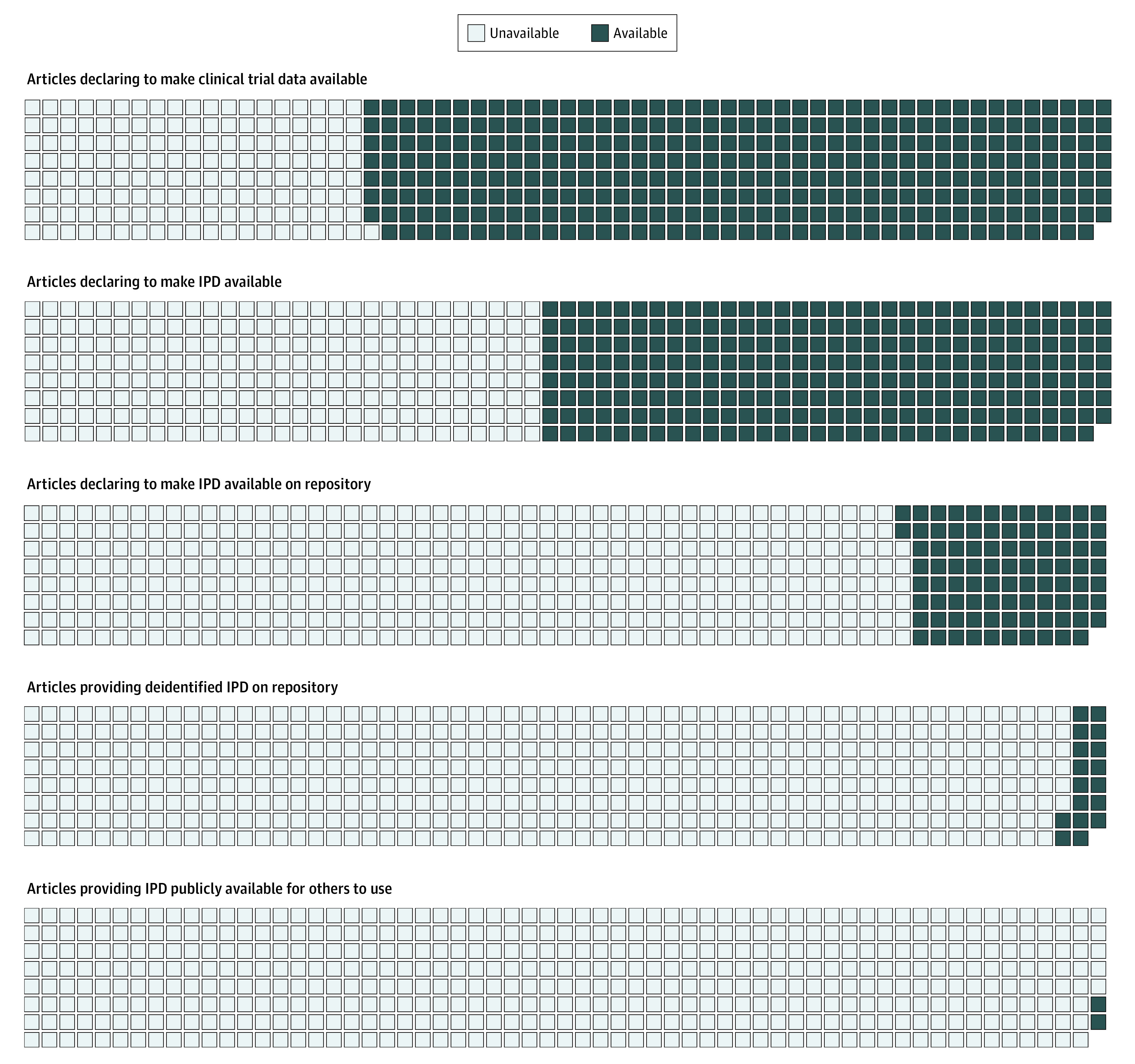
Indicators of Declared and Actual Clinical Trial Individual-Participant Data (IPD) Availability as of April 10, 2020

We compared declared with actual data availability in repositories (Table 2). Among 89 articles, information about the data was infrequently available in the repository (20 of 89 articles [22.5%]) and the data themselves were even more infrequently available there (17 of 89 articles [19%.1]) (Figure 3). Although data of NIH-funded trials (7 of 22 articles [31.8%]) were somewhat more likely to be available in repositories than data from industry-funded trials (7 of 46 articles [15.2%]), nonindustry non-NIH trials (0 of 5 articles), and trials with mixed funding (3 of 16 articles [18.8%]), data availability in repositories was not associated with type of funding (*P* = .32, Fisher exact test). Most trials provided neither information nor data in the respective repositories, mostly because of embargo and pending regulatory approval. Specifically, among the 72 articles that declared their intent but did not store data on repository, 37 (51%) made data access conditional on embargo or product approval.

**Table 2.** Availability of Individual-Participant Data in Repository by Type of Funding

Variable	Articles, No. (%)				
	Industry (n = 46)	Nonindustry NIH (n = 22)	Nonindustry, non-NIH (n = 5)	Mixed (n = 16)	Total (N = 89)
Information about data is logged in repository	9 (19.6)	7 (31.8)	1 (20.0)	3 (18.8)	20 (22.5)
Data are available on repository to request	7 (15.2)	7 (31.8)	0 (0.0)	3 (18.8)	17 (19.1)

Among reasons for data withholding, articles referred to data privacy (15 of 153 articles [9.8%]), proprietary data (14 of 153 articles [9.2%]), ongoing research (13 of 153 articles [8.5%]), and pending regulatory approval (4 of 153 articles [2.6%]). Most articles withholding data (83 of 153 articles [54.2%]) provided no reason. One article, which had no intent to make data available to others, was retracted.

## Discussion

Most trials published in *JAMA*, *Lancet*, and *NEJM* after the endorsement of the ICMJE policy declared their intent to make clinical data available. Non–industry-funded trials communicated greater intent to share data than industry-funded trials, which exhibited low declared data sharing rates even among industry funders that are members of the largest data sharing repository, the CSDR. This result is consistent with prior research on intention to share data at the ClinicalTrials.gov^37^ but departs from a trend among industry sponsors to establish mechanisms and repositories for sharing of clinical trial data.^15,17,30,31,50^

The commitment to data sharing substantially decreases when we consider indicators of actual vs declared data sharing: of 334 articles declaring that they would share data, only 2 IPD sets (0.6%; 95% CI, 0.0%-1.5%) were actually deidentified and publicly available on the journal website. Among the 89 articles declaring that they would store IPD in repositories, data from only 17 articles were found in the respective repository (Figure 3).

Although it is encouraging that data sharing appears widespread as a research norm^51^ among trialists and that repositories for secure data sharing are often considered in DSSs, the low rate of actual data availability we identified is concerning. Some data sets that are currently unavailable may simply require additional time to be released, particularly those that are associated with embargo periods, but for many unavailable data sets, actionable information about availability is lacking. This points to the need for detailed requirements that encourage authors to engage with tangible and verifiable steps toward data sharing at or before publication, including logging information about the data sets in a data repository and having time-stamped information about their planned future release. Stakeholders, including journals, funders, and research institutions, should specifically focus on policies that could narrow the wide gap we identified between declared data sharing and actual availability of clinical trial IPD.

Consistent with prior research of clinical trial data registries^30,37^ and DSSs,^7^ the language of DSSs was often ambivalent. Offering of aggregate data, collaboration demands, lengthy or unspecified embargo periods, and the use of legacy methods for access such as author or company request communicate only lukewarm commitment. Repositories can be instrumental for sharing, but real practices may diverge from intent.

The current ICMJE requirement for DSSs is restricted to clinical studies. The policy restriction may be unwarranted given the high-profile observational studies recently retracted in *Lancet*^52^ and *NEJM*^53^ because of concerns regarding data veracity and availability, pointing to potential benefits of extending data sharing requirements to observational studies.^54,55,56^

### Limitations

Our study has limitations that should be acknowledged. First, only 3 journals were considered. Moreover, we could readily investigate declared vs actual data sharing practices only for repositories. Furthermore, only 2 IPD sets were deidentified and available on journal websites, so we could not meaningfully examine the usability of shared data or reproducibility^8^ of the clinical trial studies. As more IPD sets become available, it would be interesting to assess whether they are easy to use, and how complete is the information being provided. More broadly, a repeated evaluation of data sharing intentions and practices could be valuable, particularly in the context of the ongoing clinical trial research response to the coronavirus disease 2019 pandemic that may have changed some norms and practices of clinical research.^56,57,58,59^ In addition, we determined that repeated articles from the same clinical trial have very small effect on our estimates but did not examine clustering by authors and institutions that could occur beyond the level of single trials.

## Conclusions

To promote transparency and data reuse, journals and funders should work toward incentivizing data sharing via funding mechanisms^21^ and data authorship,^23^ and simultaneously discourage ambivalent wording in DSSs and possibly mandate data sharing. They can promote the use of unique pointers to data set location in repositories and to data request forms. Standardized choices for embargo periods, access requirements, and conditions for data use as part of the data sharing process could also reduce unnecessary data withholding and turn declarative data sharing into actual transparency in clinical trial data.

## Notes

Supplement. **eTable.** Description of Clinical Trial Data Repositories **eAppendix 1. **Examples of Data Sharing Statements **eFigure.** Flow Chart of Article Eligibility **eAppendix 2.** Codebook **eAppendix 3.** Inclusion Criteria, Search Strategy, and Data Collection and Analysis
