# Supplementary material for: Evaluation of Data Sharing After Implementation of the International Committee of Medical Journal Editors Data Sharing Statement Requirement
Source: JAMA Netw Open. 2021 Jan 28;4(1):e2033972. doi: 10.1001/jamanetworkopen.2020.33972 (PMC7844597; doi:10.1001/jamanetworkopen.2020.33972)
Supplement: Supplement. — eTable. Description of Clinical Trial Data Repositories eAppendix 1. Examples of Data Sharing Statements eFigure. Flow Chart of Article Eligibility eAppendix 2. Codebook eAppendix 3. Inclusion Criteria, Search Strategy, and Data Collection and Analysis [file jamanetwopen-e2033972-s001.pdf]

Supplemental Online Content

Danchev V, Min Y, Borghi J, Baiocchi M, Ioannidis JPA. Evaluation of data sharing after implementation of the International Committee of Medical Journal Editors data sharing statement requirement. *JAMA Netw Open*. 2021;4(1):e2033972. doi:10.1001/jamanetworkopen.2020.33972

eTable. Description of Clinical Trial Data Repositories
eAppendix 1. Examples of Data Sharing Statements
eFigure. Flow Chart of Article Eligibility
eAppendix 2. Codebook
eAppendix 3. Inclusion Criteria, Search Strategy, and Data Collection and Analysis

This supplemental material has been provided by the authors to give readers additional information about their work.

© 2021 Danchev V et al. *JAMA Network Open.*

eTable. Description of Clinical Trial Data Repositories

Table S1. Description of Clinical Trial IPD Repositories[a]

Acronym / short name	Full name	URL	Host organization
CSDR	Clinical Study Date Request	https://www.clinicalstudydatarequest.com	Consortium of clinical study sponsors/funders
NCTN/NCORP Data Archive	National Clinical Trials Network (NCTN) and NCI Community Oncology Research Program (NCORP) Data Archive	https://nctn-data-archive.nci.nih.gov	National Cancer Institute (NCI)
BioLINCC	Biologic Specimen and Data Repository Information Coordinating Center	https://biolincc.nhlbi.nih.gov	National Heart, Lung, and Blood Institute (NHLBI)
YODA	The Yale University Open Data Access (YODA) Project	https://yoda.yale.edu/	Yale University
Vivli	Center for Global Clinical Research Data	https://vivli.org	The Multi-Regional Clinical Trials Center of Brigham and Women's Hospital and Harvard
DASH	NICHD Data and Specimen Hub	https://dash.nichd.nih.gov/	The Eunice Kennedy Shriver National Institute of Child Health and Human Development (NICHD)
NINDS Archived Clinical Research Datasets	NINDS Archived Clinical Research Datasets	https://www.ninds.nih.gov/Current-Research/Research-Funded-NINDS/Clinical-Research/Archived-Clinical-Research-Datasets	National Institute of Neurological Disorders and Stroke (NINDS)
BORIS	Bern Open Repository and Information System	https://boris.unibe.ch/	University of Bern and the Bern University Hospital
NIDDK Central Repository	NIDDK Central Repository	https://repository.niddk.nih.gov	National Institute of Diabetes and Digestive and Kidney Diseases (NIDDK)
JCHR	JAEB Center for Health Research	https://www.jaeb.org/	JAEB Center for Health Research

[a] Included are only repositories that were referenced in two or more data sharing statements.

eAppendix 1. Examples of Data Sharing Statements

Matta MK, Zusterzeel R, Pilli NR, et al. Effect of sunscreen application under maximal use conditions on plasma concentration of sunscreen active ingredients. *JAMA*. doi:10.1001/jama.2019.5586

Data Sharing Statement

Data
Data available: Yes
Data types: Deidentified participant data
How to access data: Deidentified participant data are provided as an online supplement to the manuscript.
When available: With publication

Supporting Documents
Document types: .csv file; see Supplement 3

Additional Information
Who can access the data: Everyone
Types of analyses: For any purpose
Mechanisms of data availability: No restrictions
Any additional restrictions: None

Data Sharing Statement

Fleischer. Effect of Epicutaneous Immunotherapy vs Placebo on Reaction to Peanut Protein Ingestion Among Children With Peanut Allergy. *JAMA*. Published February 22, 2019. 10.1001/jama.2019.1113

Data
Data available: No

Data Sharing Statement

Sparano JA, Gray RJ, Ravdin PM, et al. Clinical and Genomic Risk to Guide the Use of Adjuvant Therapy for Breast Cancer. N Engl J Med. DOI: 10.1056/NEJMoa1904819.

Question	Authors' Response
Will the data collected for your study be made available to others?	Yes
Would you like to offer context for your decision?	—
Which data?	Complete de-identified patient data set
Additional information about data	De-identified data set via NCI data repository https://nctn-data-archive.nci.nih.gov
How or where can the data be obtained?	https://nctn-data-archive.nci.nih.gov Contact: For questions about the NCTN/NCORP Data Archive, please contact NCINCTNDataArchive@mail.nih.gov (link sends e-mail).
When will data availability begin?	with publication
When will data availability end?	—
Will any supporting documents be available?	—
Which supporting documents?	—
Additional information about supporting documents	—
How or where can supporting documents be obtained?	—
When will supporting documents availability begin?	—
When will supporting documents availability end?	—
To whom will data be available?	NCI has created a centralized, controlled-access database, called the NCTN/NCORP Data Archive, for storing and sharing datasets generated from clinical trials of the National Clinical Trials Network (NCTN) to make these datasets available in a timely manner, on appropriate terms and conditions, to researchers who wish to analyze the data in secondary studies to enhance the public health benefit of the original work. If imaging data are available as part of a trial, a link will be provided to The Cancer Imaging Archive

Data Sharing Statement

Stone GW, Kappetein AP, Sabik JF, et al. Five-Year Outcomes after PCI or CABG for Left Main Coronary Disease. N Engl J Med. DOI: 10.1056/NEJMoa1909406.

Question	Authors' Response
Will the data collected for your study be made available to others?	No
Would you like to offer context for your decision?	—
Which data?	—
Additional information about data	—
How or where can the data be obtained?	—
When will data availability begin?	—
When will data availability end?	—
Will any supporting documents be available?	—
Which supporting documents?	—
Additional information about supporting documents	—
How or where can supporting documents be obtained?	—
When will supporting documents availability begin?	—
When will supporting documents availability end?	—
To whom will data be available?	—
For what type of analysis or purpose?	—
By what mechanism?	—
Any other restrictions?	—
Additional information	—

This statement was posted on September 28, 2019, at NEJM.org.

© 2021 Danchev V et al. *JAMA Network Open.*

Data Sharing Statement

Llanos-Cuentas A, Lacerda MVG, Hien TT, et al. Tafenoquine versus Primaquine to Prevent Relapse of *Plasmodium vivax* Malaria. N Engl J Med. DOI: 10.1056/NEJMoa1802537.

Question	Authors' Response
Will the data collected for your study be made available to others?	Yes
Would you like to offer context for your decision?	No
Which data?	Anonymized individual participant data, the annotated case report form, protocol, reporting and analysis plan, data set specifications, raw data set, and analysis-ready data set
Additional information about data	No
How or where can the data be obtained?	Proposals should be submitted to www.clinicalstudydatarequest.com
When will data availability begin?	Within 6 months of this publication
When will data availability end?	N/A
Will any supporting documents be available?	Yes
Which supporting documents?	Protocol, reporting and analysis plan, data set specifications, and clinical study report
Additional information about supporting documents	No
How or where can supporting documents be obtained?	Proposals should be submitted to www.clinicalstudydatarequest.com
When will supporting documents availability begin?	Within 6 months of this publication
When will supporting documents availability end?	N/A
To whom will data be available?	For research proposals approved by an independent review committee
For what type of analysis or purpose?	For research proposals approved by an independent review committee
By what mechanism?	Proposals should be submitted to www.clinicalstudydatarequest.com
Any other restrictions?	A data access agreement will be required
Additional information	No

This statement was posted on January 17, 2019, at NEJM.org.

SPRINT MIND Investigators for the SPRINT Research Group. Effect of intensive vs standard blood pressure control on probable dementia: a randomized clinical trial [published online January 17, 2019]. doi:10.1001/jama.2018.21442

Data Sharing Statement

Data
Data available: Yes
Data types: Deidentified participant data, Data dictionary
How to access data: Data will be available in BioLINCC: https://biolincc.nhlbi.nih.gov/studies/sprint
When available: beginning date: 07-01-2020

Supporting Documents
Document types: None

Additional Information
Who can access the data: Data will be available for investigators providing an IRB/Ethics approval or certification of exemption from IRB/Ethics review, and also agreeing to the terms and conditions of a data use agreement
Types of analyses: Data will be available for any purpose, unless prohibited by the informed consent.
Mechanisms of data availability: Data will be available for investigators providing an IRB/Ethics approval or certification of exemption from IRB/Ethics review, and also agreeing to the terms and conditions of a data use agreement.

© 2019 American Medical Association. All rights reserved.

© 2021 Danchev V et al. *JAMA Network Open.*

eFigure. Flow Chart of Article Eligibility

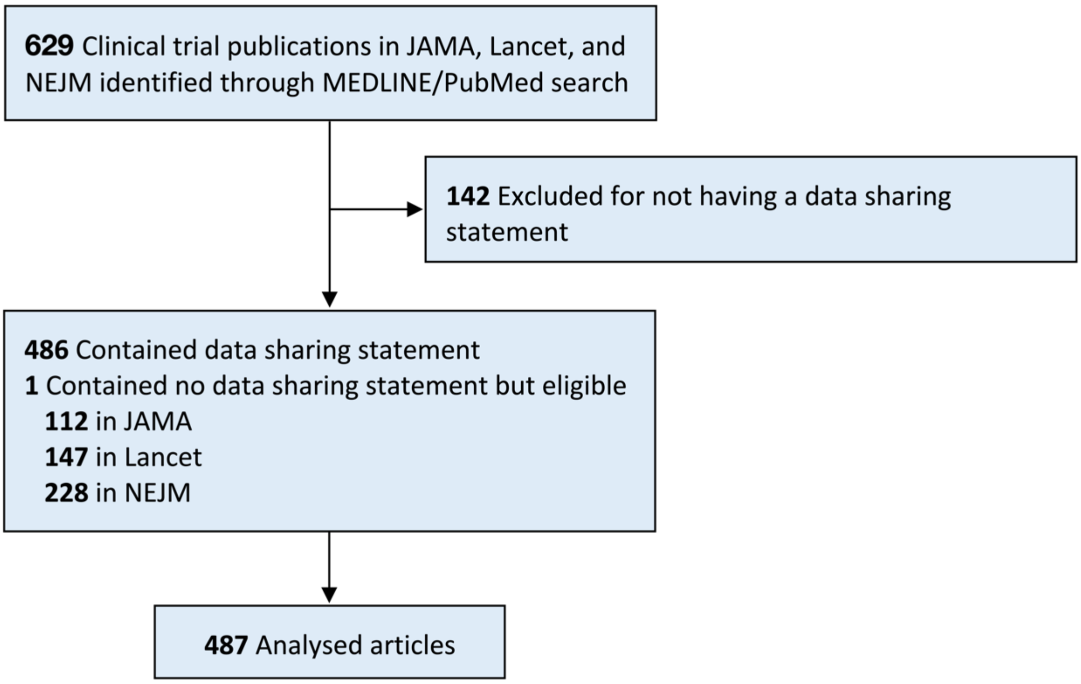

© 2021 Danchev V et al. *JAMA Network Open.*

eAppendix 2. Codebook

A. Declared data sharing

1 = YES → Code **B**, **C**, **D**, and **F**

0 = NO → Code **E** and **F**

B. Type of declared available data [1 if applicable, blank otherwise]

Deidentified individual participant data
Aggregate data only
Unspecified/ partial data

C. Access to data [1 if applicable, blank otherwise]

Request to authors
Request to committee/ group/ unit
Request to company
Request to repository/ archive
  Name of repository [Name as noted on DSS]
  Repository contains information about the data/study [1 = Yes, 0 = No]
  Data is available on repository [1 = Yes, 0 = No]
Access unspecified
Data is available to others

D. Conditions for data sharing [1 if applicable, blank otherwise]

Embargo
  Embargo period [in Months]
  If embargo period unspecified, copy wording from the DSS
Collaboration
Product approval

E. Reasons for why data not available [1 if applicable, blank otherwise]

No reason given
Data privacy
Time and cost
Ongoing trial/ research
Regulatory approval
Proprietary data
Shared among co-investigators only
Data may be available for collaboration
Data may be available upon request

F. Funder type*

1 = Industry
2 = Non-industry NIH

© 2021 Danchev V et al. *JAMA Network Open.*

3 = Non-industry non-NIH
4 = Mixed (any combination of industry, non-industry NIH, non-industry non-NIH)

* Only funding is eligible (in-kind support or supplies provided at no charge are not eligible).

© 2021 Danchev V et al. *JAMA Network Open.*

eAppendix 3. Inclusion Criteria, Search Strategy, and Data Collection and Analysis

Inclusion and exclusion criteria

In order to be eligible to be selected in the study, a published paper must meet the following inclusion criteria:

1. Publication reports clinical trial results
2. Published in JAMA, NEJM, Lancet
3. Published since July 1, 2018
4. Type of publication is Article
5. Contain a Data Sharing Statement (DSS)*

*Articles published in 2020 that meet criteria 1 to 4 are eligible even if no DSS is present as these are likely submitted after July 1, 2018, and therefore fall under the requirements of the ICMJE data sharing policy.

Excluded from the study were publications that contain no Data Sharing Statement due to:

1. Submission prior to July 1, 2018
2. Study not a clinical trial (e.g., observational)
3. Type of publication is letter/correspondence

Search strategy

A MEDLINE/PubMed search was performed on April 4, 2020 using the following search strategy:

(((("The New England journal of medicine"[Journal]) OR "JAMA"[Journal]) OR "Lancet (London, England)"[Journal]) AND clinical trial[Publication Type]) AND ("2018/07/01"[Date - Publication] : "2020/04/04"[Date - Publication])

As of April 04, 2020, the search yielded 629 results. Out of those, 486 publications were clinical trials and contained a DSS. One 2020 publication met 1 to 4 inclusion criteria but contained no DSS and was included in the study sample as not sharing data because articles published in 2020 were likely submitted after policy's effective date, July 1, 2018. We conducted a cross-sectional observational study of the resulting sample of 487 articles.

Independent review of articles

For each of the 487 articles, two reviewers independently evaluated the Data Sharing Statement and funding statement, using procedures described in eAppendix 2. Discrepancies were resolved unanimously or by a third reviewer.

© 2021 Danchev V et al. *JAMA Network Open.*
